# The mitochondrial genome of *Cavia aperea*

**DOI:** 10.1080/23802359.2020.1768918

**Published:** 2020-05-22

**Authors:** Azizia Wahedi, Anja Günther, Alexandra Weyrich, Neal Sondheimer

**Affiliations:** a Program in Genetics and Genome Biology, The Hospital for Sick Children, Toronto, Canada; b The Department of Paediatrics, The Hospital for Sick Children, Toronto, Canada; c Max Planck Research Group Behavioural Ecology of Individual Differences, Max Planck Institute for Evolutionary Biology, Plön, Germany; d Leibniz Institute for Zoo and Wildlife Research (IZW), Berlin, Germany

**Keywords:** mtDNA, guinea pig

## Abstract

*Cavia aperea* is a wild guinea pig found throughout South America. The previously published mitochondrial sequence for *C. aperea* was highly divergent from the *C. porcellus* sequence and contained stop codons within open reading frames. Here we resequenced the mitochondrial genomes of *C. aperea* and *C. porcellus*. Both sequences reflect gene organization typical for mammalian mitochondrial DNA. Our *C. aperea* mtDNA sequence shows that all of the open reading frames are intact, but confirms the strikingly low level of sequence identity (92.7%) with the closely related *C. porcellus* mtDNA.

*Cavia aperea* is a wild guinea pig found through large parts of South America (Rood 1972). Allometric studies confirm that the laboratory strain *Cavia porcellus* descended from *C. aperea* (Kruska and Steffen 2013). Both *C. aperea* and *C. porcellus* have been used in behavioral research and they may provide a superior model for reproductive research to mouse because of similarities between progesterone levels in pregnancy in humans and in cavies and the potential for preterm delivery (Mitchell and Taggart 2009). Hybrids between *aperea* and *porcellus* have been generated for studies of chromosome structure (George et al. 2009), reproduction (Rood and Weir 1970) and enzymology (Carter et al. 1972). The hybrids are both viable and fertile.

The mitochondrial sequence for *C. aperea* was previously published (Cao and Xia 2015) based upon reads from a whole genome sequencing study (Weyrich et al. 2014). The sequence (KT439327) was highly divergent from the sequence of *porcellus* mtDNA. This was surprising, because mitochondrial genetic incompatibility can prevent hybridization and lead to male sterility, colorfully known as ‘mother’s curse’ (Wolff et al. 2014). However the published *aperea* sequence KT439327 also contained nonsense codons in open reading frames suggesting the possibility of error. Here we re-sequenced the mtDNA of *C. aperea* and *C. porcellus*.

*C. aperea* were collected in Uruguay and were housed at the University of Bielefeld Department Of Animal Behavior. *C. porcellus* were obtained from Charles River. DNA was amplified using primers (available upon request) based upon the published sequences. Amplified sequences were confirmed by gel electrophoresis, sequenced and assembled (Geneious Prime, Biomatters Ltd.).

The mtDNA sequence of *Cavia aperea* (MT017566) and *Cavia porcellus* (MT017565) have been deposited in GenBank. The sequenced DNA is stored at the SickKids Central Biobank (Canadian Tissue Repository Network #BRC-00181) as S01-565 (*porcellus*) and S01-566 (*aperea*). We constructed a neighbour-joining phylogenetic tree of these sequences (Figure 1). MT017565 is highly similar to the published sequence NC_000884.1 with no differences in amino acid sequence and only six non-coding substitutions in total. Our *aperea* mtDNA is 16,681 nucleotides in length. The sequence confirms that spurious insertions were present in KT439327. The overall gene structure of the *C. aperea* mtDNA is unremarkable, with no change in the canonical ordering or orientation of the coding genes. Like the previously published sequence of *aperea*, we found a low level of sequence identity to *porcellus* (92.7%). Amino acid differences were found in 12 out of the 13 protein coding genes including the following, ND1 (3), ND2 (14), ND3 (8), ND4 (15), ND5 (37), ND6 (9), ATP6 (9), ATP8 (7), COX1 (5), COX2 (4), COX3 (4), and CYTB (10) with the number in parentheses indicating the number of residue changes.

**Figure 1.**
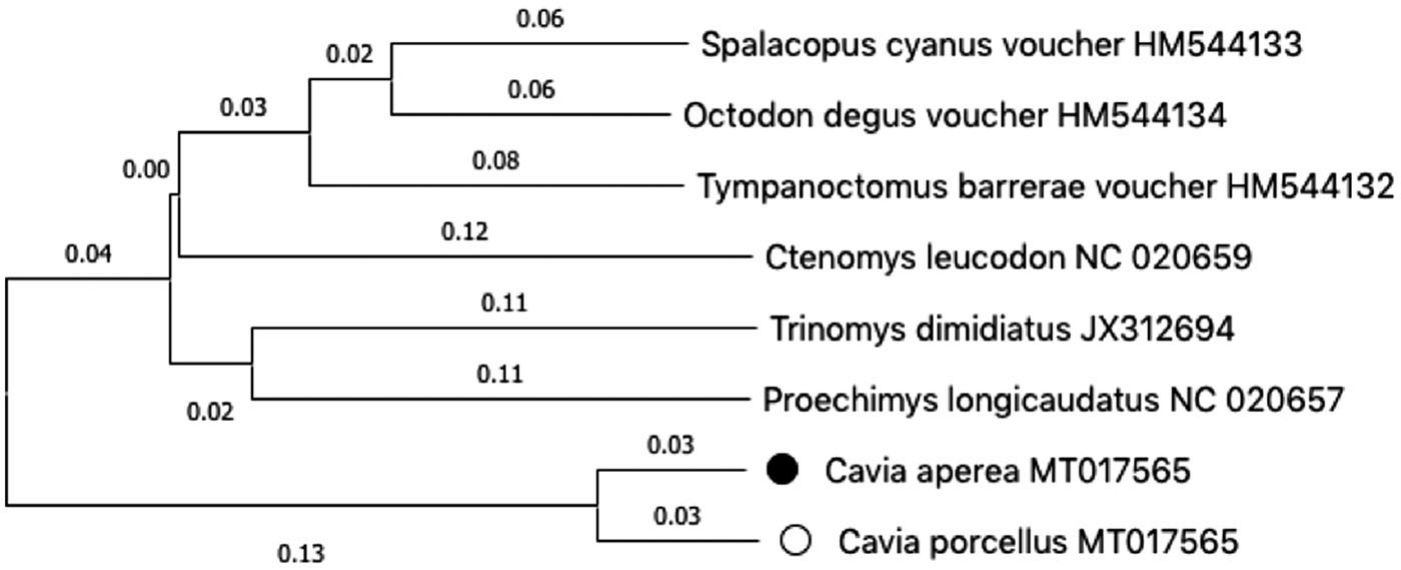
Phylogenetic relationship of the *Cavia aperea* ● and *Cavia porcellus* ^, and six other rodent species. Tree was constructed using the ClustalW alignment and neighbour-joining analysis in Mega X (Kumar et al. 2018).

This level of sequence divergence is striking. For example, the identity between the human reference sequence (rCRS) and the sequence of Neandertal mtDNA is 98.7% (Green et al. 2008) and the maximal known divergence between two modern human mtDNAs is 99.4% (Ingman et al. 2000). The low level of identity between *cavia* and *aperea* mtDNA is remarkable given their capacity to interbreed. The ability to survive the high level of incompatibility between mitochondrial and nuclear genomes requires further investigation.

## Data Availability

The data that support the findings of this study are openly available on GenBank using the accession numbers MT017566 (https://www.ncbi.nlm.nih.gov/nuccore/MT017566) and MT017565 (https://www.ncbi.nlm.nih.gov/nuccore/MT017565).
